# Effect of Divalent Cations (Cu, Zn, Pb, Cd, and Sr) on Microbially Induced Calcium Carbonate Precipitation and Mineralogical Properties

**DOI:** 10.3389/fmicb.2021.646748

**Published:** 2021-04-08

**Authors:** Yumi Kim, Sunki Kwon, Yul Roh

**Affiliations:** Department of Earth and Environmental Sciences, Chonnam National University, Gwangju, South Korea

**Keywords:** urea hydrolysis, *Sporosarcina pasteurii*, bioremediation, heavy metals, bio-co-precipitation

## Abstract

Microbially induced calcium carbonate precipitation (MICP) is a bio-geochemical process involving calcium carbonate precipitation and possible co-precipitation of other metals. The study investigated the extent to which a urease-positive bacterium, *Sporosarcina pasteurii*, can tolerate a range of metals (e.g., Cu, Zn, Pb, Cd, and Sr), and analyzed the role of calcium carbonate bioprecipitation in eliminating these divalent toxicants from aqueous solutions. The experiments using *S. pasteurii* were performed aerobically in growth media including urea, CaCl_2_ (30 mM) and different metals such Cu, Zn, Pb, and Cd (0.01 ∼ 1 mM), and Sr (1 ∼ 30 mM). Microbial growth and urea degradation led to an increase in pH and OD_600_, facilitating the precipitation of calcium carbonate. The metal types and concentrations contributed to the mineralogy of various calcium carbonates precipitated and differences in metal removal rates. Pb and Sr showed more than 99% removal efficiency, whereas Cu, Zn, and Cd showed a low removal efficiency of 30∼60% at a low concentration of 0.05 mM or less. Thus the removal efficiency of metal ions during MICP varied with the types and concentrations of divalent cations. The MICP in the presence of divalent metals also affected the mineralogical properties such as carbonate mineralogy, shape, and crystallinity.

## Introduction

Heavy metal and radionuclide pollution in subsurface environments is a serious environmental concern. Conventional remediation techniques have been utilized recently to detoxify these metals from contaminated environments via chemical precipitation, evaporation, electrochemical treatment, oxidation/reduction, carbon adsorption, ion exchange, membrane filtration, and reverse osmosis (Mugwar and Harbottle, 2016). Microbially induced calcium carbonate precipitation (MICP), however, is a bio-geochemical process involving precipitation of calcium carbonate and possible co-precipitation of heavy metals and radionuclides (Fujita et al., 2000). The toxic metals are removed via direct precipitation leading to metal carbonate formation, or by co-precipitation incorporating metals such Cu^2+^, Cd^2+^, Zn^2+^, Pb^2+^, and Fe^2+^ in the lattice structure of calcium carbonate via substitution of Ca^2+^ (Kang et al., 2014b; Krajewska, 2018). In addition, the mechanisms underlying metal incorporation into biogenic calcium carbonate suggest that biogenic calcium carbonate particles are typical porous materials with large specific surface area, which strongly enhances metal absorption (Zhu et al., 2016; Qian et al., 2017). Thus the emergence of MICP as a promising in-situ remediation technology has led investigators to explore the use of microorganisms in the removal of toxic metals and radionuclides effectively. Li et al. (2013) demonstrated high removal rates (ranging from 88% to 99%) of Ni, Cu, Pb, Co, Zn, and Cd within 48 h using metal-resistant strains via MICP. However, the removal rate may vary depending on the initial concentration of metal ions, and a low metal concentration contributes to high efficiency. Mugwar and Harbottle (2016) analyzed the correlation between the growth of microorganisms and the removal of metal ions at various concentrations. The results demonstrated that elevated pH and calcium precipitation were strongly linked to the removal of Zn and Cd, but only partially affected the elimination of Pb and Cu (Mugwar and Harbottle, 2016).

Although many studies have reported the removal efficiency of metal ions via MICP, the emphasis was mainly on the role of microbial growth and urease activity in the removal of metal ions rather than carbonate mineral formation and mineralogical characteristics (Li et al., 2013; Agarwal and Singh, 2017; Bhattacharya et al., 2018). During MICP, carbonate crystals were formed various anhydrous calcium carbonate minerals such as calcite (β-CaCO_3_), aragonite (λ-CaCO_3_) and vaterite (μ-CaCO_3_), as well as other crystalline hydrated phases such as monohydrocalcite (CaCO_3_⋅H_2_O) and hexahydrocalcite (CaCO_3_⋅6H_2_O), in addition to amorphous calcium carbonate (Costa et al., 2017; Chaparro-Acuña et al., 2018). The morphology and polymorphism of biogenic calcium carbonates suggest microbial strain-specific mineralization associated with various biomacromolecular templates (Rodriguez-Navarro et al., 2012; Cao et al., 2016; Kim and Roh, 2019). However, the mechanisms underlying the effects of biomolecular and metal co-precipitation on carbonate composition, morphology, and size, have yet to be elucidated. Therefore, the objectives of this study were to investigate the extent to which a urease-positive bacterium, *Sporosarcina pasteurii*, can tolerate a range of metals (e.g., Cu, Zn, Pb, Cd, and Sr). The study also analyzes the removal efficiency of these metallic toxicants via calcium carbonate precipitation and their effects on mineralogical properties of the precipitated crystals.

## Materials and Methods

### Microorganisms and Culture Media

*Sporosarcina pasteurii*, a gram-positive, facultative anaerobe and endospore-forming bacterium, formerly known as *Bacillus pasteurii*, was cultured (Warren et al., 2001). *S. pasteurii* (KCTC 3558) was obtained from Korean Collection for Type Culture (KCTC, Daejeon, South Korea). The growth media modified from the previous method (Gorospe et al., 2013; Kang and Roh, 2017; Kim and Roh, 2019) for *S. pasteurii* cultivation and carbonate precipitation contained the following ingredients: 10 g/L yeast extract, 5 g/L proteose peptone, 1 g/L glucose, and 24 g/L NaCl. The medium was autoclaved at 121°C and 1.2 kgf/cm^2^ for 20 min. The pH of the medium was about 7. The strain was cultivated in the medium modified with 0.2 μm filter-sterilized 20 g/L urea under aerobic conditions at 25°C for 7 days.

### Bioprecipitation of Heavy Metals and a Radionuclide

During the precipitation of calcium carbonate minerals induced by ureolytic bacteria, metal cations can be substituted for Ca ions in the lattice structure of the carbonate minerals (Eq. 1) (Wu et al., 2011).

(1) $$\text{xM}e^{2}+\left(1-\text{x}\right)\text{C}a^{2}+2\text{H}CO_{3}^{-}↔\text{C}a_{\left(1-x\right)}\text{M}e_{x}\text{C}O_{3}+{\text{H}}_{2}\text{O}+\text{C}O_{2}$$

In the above equation, *Me^2+^* denotes the metallic cation.

Co-precipitation of heavy metals or a radionuclide was induced with calcium carbonate by injecting five different toxic ions including Cu, Zn, Pb, Cd, and Sr into the microbial growth media containing urea and calcium ions. The stock solution of 1 M calcium chloride (CaCl_2_⋅2H_2_O, MW: 147.01) was sterilized by autoclaving, followed by the addition of the solution to all experimental tubes at a concentration of 30 mM. Stock solutions of heavy metals were prepared by dissolving copper chloride (CuCl_2_⋅2H_2_O, MW: 170.48), zinc chloride (ZnCl_2_, MW: 136.30), lead acetate trihydrate [Pb (CH_3_CO_2_) _2_⋅3H_2_O, MW: 379.33] and cadmium acetate [Cd (C_2_H_3_O_2_)_2_⋅2H_2_O, MW: 266.53] in distilled water. The radionuclide solution was prepared using strontium chloride hexahydrate (SrCl_2_⋅6H_2_O, MW: 266.62) instead of Sr^90^. Bio-co-precipitation experiments were performed in 50 mL conical tubes. To determine the metal or radionuclide removal, metal ions including Cu, Zn, Pb, and Cd were injected into 30 mL aliquots of microbial media at concentrations of 0.01, 0.05, 0.1, 0.5, and 1 mM, respectively. However, Sr as a radionuclide with relatively low microbial toxicity was injected into the medium at 1, 5, 10, 20 and 30 mM. Microbial co-precipitation was induced via addition of 0.3 mL of enriched bacterial culture (1% v/v) to the metal or radionuclide-containing media. All the tubes were shaken at 150 rpm to ensure complete mixing at room temperature (25°C) for 14 days. During experiments, 5 mL of suspension was withdrawn with a 6 mL syringe at 1, 3, 7, and 14 days to determine the changes in pH, optical density (OD_600_), and the concentration of the residual metal or radionuclide in the media. Also, the mineralogical properties of the precipitates recovered from the withdrawn samples were analyzed.

### Analytical Methods

The mineral formation was correlated with bacterial growth by measuring the changes in pH and optical density (OD_600_) in the media during the experiments. Using a 0.2 μm syringe-filter, 5 mL aliquots of suspension obtained after 0, 1, 3, 7, and 14 days were filtered and sub-divided for analysis. The pH was measured using a HM-25R pH meter (DKK-TOA, Tokyo, Japan) and the optical density of biomass was measured via UV-vis absorption at 600 nm using a UV-1650PC spectrophotometer (Shimadzu, Kyoto, Japan). Concentrations of toxic metals and calcium ions were determined via inductively coupled plasma-atomic emission spectroscopy (ICP-AES) and -mass spectrometry (ICP-MS) with Nexion 2000 (Perkin-Elmer, Waltham, MA, United States) using the standard method. Mineralogical characteristics of the MICP were examined via X-ray diffraction (XRD) and field emission scanning electron microscopy (FE-SEM) with energy dispersive X-ray (EDS) analyses. The precipitates for XRD and SEM analyses were collected by centrifuging the suspension at 3,000 rpm for 5 min, and then separated from the supernatants. The precipitates in the conical tube were carefully rinsed with distilled water twice, and then dried in an oven at 60°C to obtain calcium carbonate in the solid phase. XRD analysis was performed using an Empyrean X-Ray Diffractometer (Panalytical, Almelo, Netherlands) equipped with Cu Kα radiation (40 kV, 30 mA) at a scan speed of 5 θ/min. The FE-SEM analysis was conducted using Hitachi S-4700 FE-SEM (Hitachi, Tokyo, Japan) at an accelerating voltage of 15–20 kV with EDS (Phillips, Eindhoven, Netherlands) to determine the morphology and elemental composition of the calcium carbonates. The elemental composition and adsorption state of metals on and near the surface of carbonate minerals were investigated via X-ray photoelectron spectroscopy (XPS) using VG Multilab 2000 (VG Systems, East Grinstead, United Kingdom).

## Results

### Microbial Growth and pH

**FIGURE 1:**
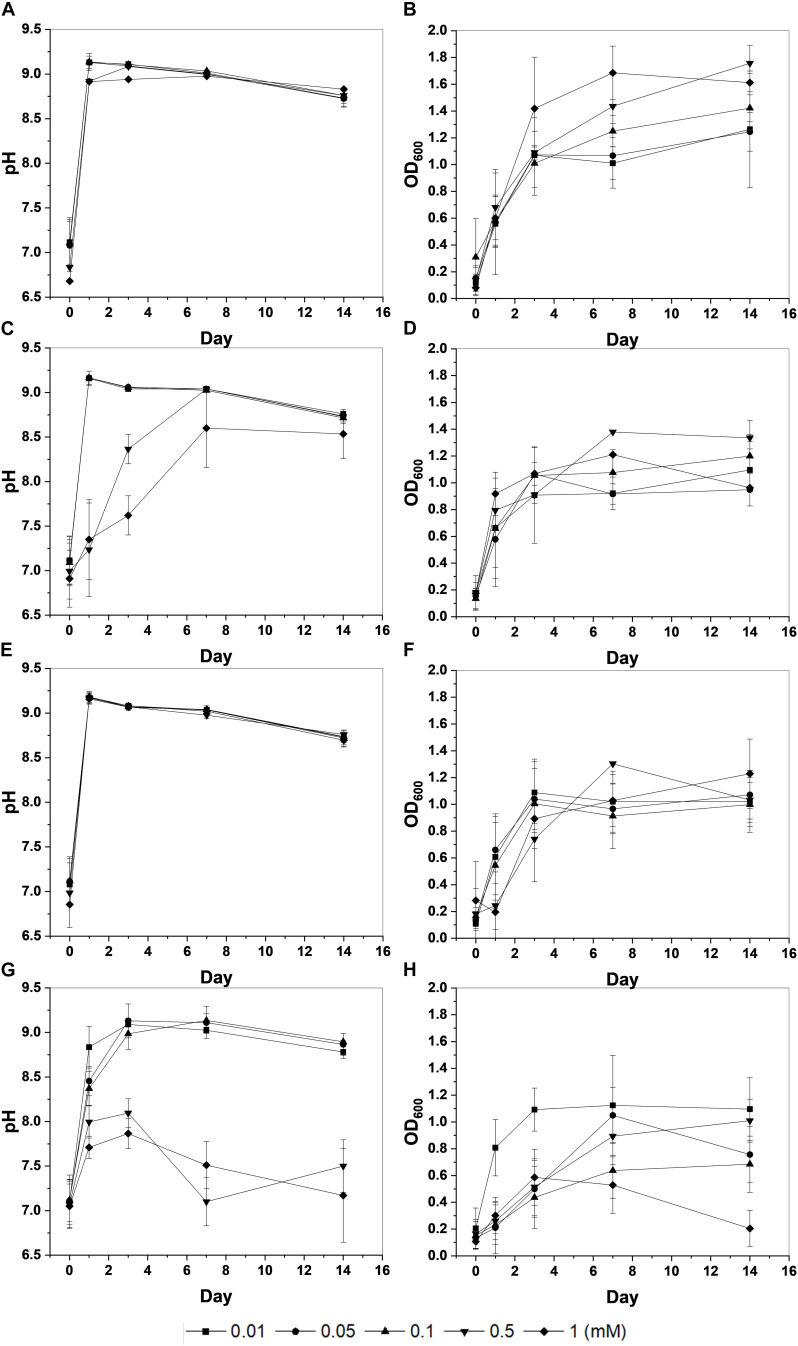
Changes in pH and optical density (OD_600_) of KCTC 3558 culture at various metal concentrations (mM): Cu **(A,B)**; Zn **(C,D)**; Pb **(E,F)**; and Cd **(G,H)**.

During the microbial growth, the strain *S. pasteurii* KCTC 3558 hydrolyzed urea with ammonium to increase the pH of the medium. Biomass (OD_600_) and pH changes showed that Cd had the highest toxicity for the strain among the heavy metals investigated (Figure 1). The microorganism had an OD_600_ value of around 1 (approx. 10^7^/mL of cells) within 3 days under most metal concentrations, whereas Cd-containing experiments yielded a low OD_600_ value except for the lowest concentration of 0.01 mM. Microbial growth also increased the pH of the medium, similarly. The pH value of the medium displaying active growth of microorganisms increased to 9 within 3 days. However, high concentrations of Cu (1 mM) and Zn (0.5 to 1 mM) slowed the increase in pH, and the injection of Cd at 0.5 to 1 mM did not increase the pH, indicating the inhibition of microbial growth.

### Metal Removal During Calcium Carbonate Precipitation

Precipitation of carbonate minerals containing the following divalent cations by *S. pasteurii* KCTC 3558 was determined via chemical and mineralogical analyses.

### Copper

**FIGURE 2:**
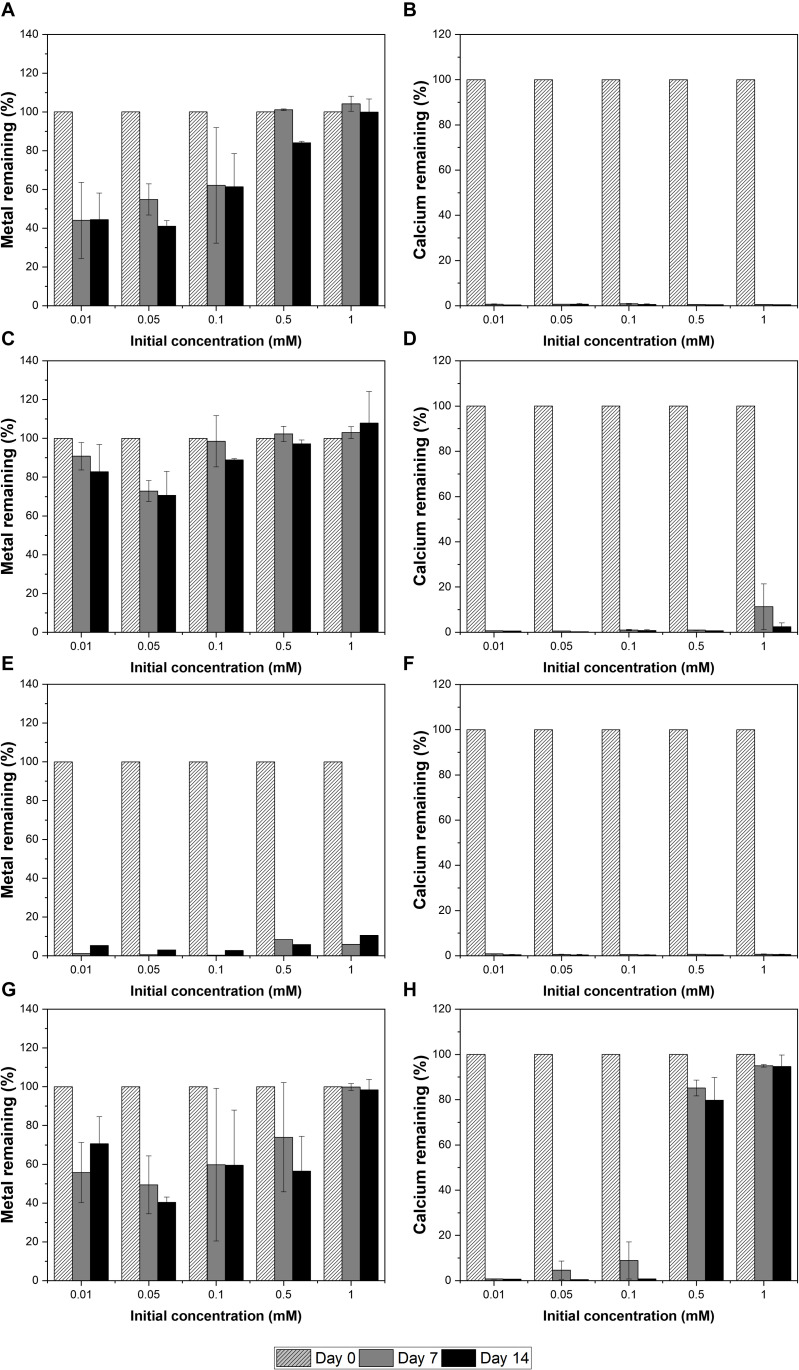
Removal of metals and calcium at a range of metal concentrations by KCTC 3558 in urea growth medium for 2 weeks: Cu **(A,B)**; Zn **(C,D)**; Pb **(E,F)**; and Cd **(G,H)**.

**FIGURE 3:**
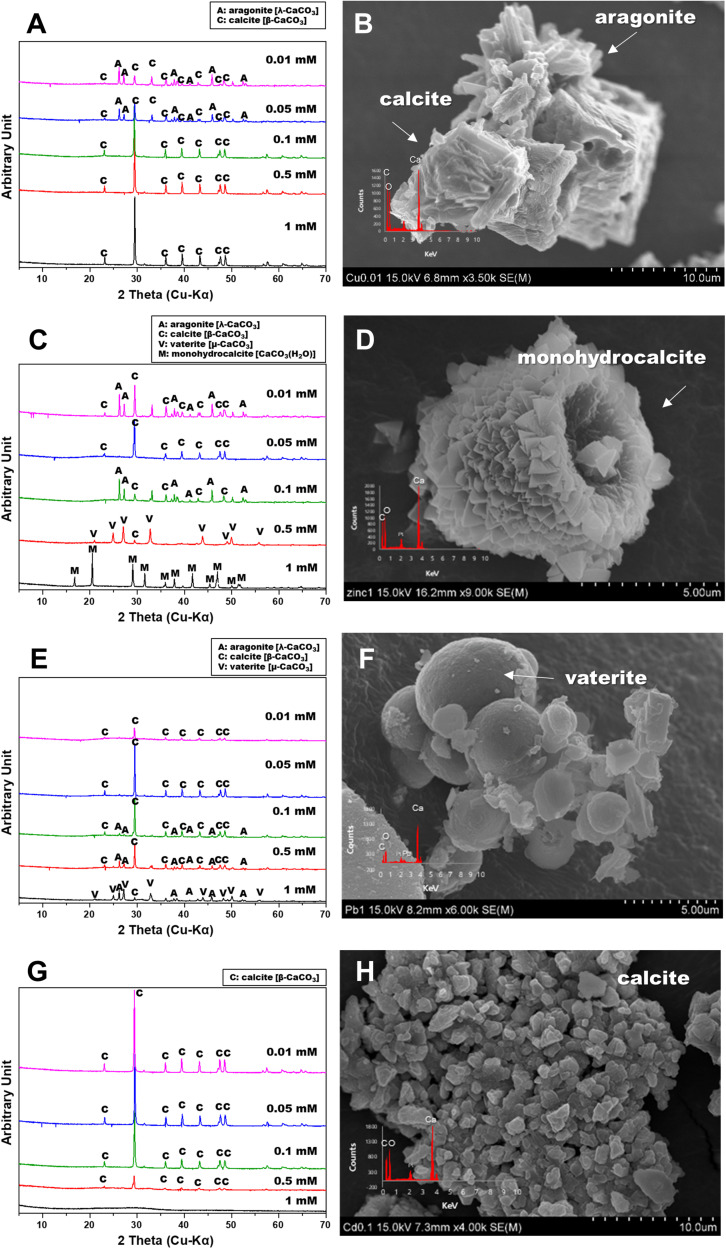
X-ray diffraction spectra and scanning electron microscopy images of calcium carbonate precipitated from various metals: Cu **(A,B)**; Zn **(C,D)**, Pb **(E,F)**, and Cd **(G,H)**.

The strain was actively grown in the medium containing Cu and induced calcium carbonate precipitation. The values of OD_600_ and pH increased rapidly except for 1 mM Cu concentration corresponding to the removal of Cu and Ca. Removal of Cu according to the growth of microorganisms was effective at concentrations less than 0.05 mM Cu and showed a removal rate of up to 60% after 2 weeks (Figure 2A). However, as the Cu concentration increased, the removal efficiency tended to decrease. In the presence of 1 mM Cu, there was little change in concentration for 2 weeks, indicating that no Cu was removed by microorganisms or carbonate precipitation. The higher the Cu, the lower was the removal efficiency, but the residual concentration of Ca in the medium was less than 1% (Figure 2B). This result indicated that the carbonate ions (CO_3_^2–^) generated due to microbial growth and ureolysis reacted with Ca to induce calcium carbonate precipitation and were not affected by the concentration of coexisting Cu ions. Analysis of the mineralogical properties of the precipitates recovered after 2 weeks of microbial reaction established the formation of calcium carbonate at all Cu concentrations (Figure 3A). However, aragonite and calcite were the calcium carbonates formed in the medium containing low concentrations of Cu (0.01 to 0.05 mM). Aragonite and calcite showed similar composition of Ca, C, and O, but the morphology differed due to crystal structure. Figure 3B shows needle-shaped aragonite and rhombohedral calcite structures. Only calcite was formed at a Cu concentration of 0.1 to 1 mM, but the morphology observed by SEM analysis varied (Supplementary Figure 1). Calcium carbonate formed with 0.1 mM Cu showed aggregates of numerous hexagonal prism-shaped particles (Supplementary Figure 1A), whereas rod-shaped crystals were mainly observed in calcium carbonate formed at 1 mM Cu (Supplementary Figure 1B).

### Zinc

The removal of Zn was not effective during the microbial precipitation of carbonate minerals. In contrast to the high removal efficiency of Cu at low concentration, 70% of the initial Zn concentration still remained after 2 weeks of microbial reaction in the medium even at 0.01 to 0.05 mM (Figure 2C). Zn concentration of 0.5 mM or higher in the medium had a substantially lower removal rate compared with the complete removal of Zn below 0.5 mM reported previously (Mugwar and Harbottle, 2016). In the presence of Zn concentration of 0.5 and 1 mM, the increase in pH to 8.5 or higher occurred in about 7 days, indicating slow urea decomposition by microorganisms (Figure 1C). Excluding 0.5 mM and 1 mM Zn concentration, the value of OD_600_ reached 1.0 within 7 days (Figure 1D). However, more than 99% Ca in the medium was removed within 2 weeks at all concentrations regardless of the Zn removal rate and precipitated as calcium carbonate (Figure 2D). XRD analysis showed calcite formation in a Zn concentration range of 0.01 mM to 0.5 mM, accompanied by aragonite formation at 0.01 mM and 0.1 mM of Zn, and vaterite at 0.5 mM of Zn (Figure 3C). The inclusion of 1 mM of Zn yielded only precipitates of monohydrocalcite. Morphological analysis of minerals via SEM revealed the formation of rhombohedral calcite and aragonite aggregates in the form of needles in the presence of 0.01 mM Zn, and vaterite formed in the presence of 0.5 mM Zn showed a typical spherical shape (Supplementary Figures 2A,B). Monohydrocalcite formed aggregates of dipyramidal crystals (Figure 3D). According to Hausinger (2004), Zn ions influenced the formation of monohydrocalcite and vaterite during the biological precipitation of calcium carbonate (Hausinger, 2004). These results suggest that high concentrations of Zn (0.5 mM or higher) inhibited the growth of microorganisms and delayed the rate of increase in OD_600_ and pH, but did not affect the precipitation of calcium carbonate in the media containing Ca ions for 2 weeks. In addition, the effect of co-precipitation or Zn adsorption in calcium carbonate crystals during this process was not clear. However, it was found to affect the type of calcium carbonate precipitated microbially via formation of monohydrate.

### Lead

In the presence of Pb up to 1 mM, the active growth of microorganisms corresponded to an increase in OD_600_ value and resulted in a large increase in pH (Figures 1E,F). Unlike other metals, the increase in Pb concentration had little effect on microbial growth and the rate of pH change. During MICP, Pb was removed by an average of 94.5% and calcium by an average of more than 99% (Figures 2E,F). However, the results of XRD and SEM analysis indicated that the removal of metals and calcium from the medium was not completely altered by the precipitation of calcium carbonate (Figures 3E,F). Inclusion of 0.01 mM Pb led to the formation of mainly amorphous Ca-precipitates and a small amount of calcite (Figure 3E). However, inclusion of 0.05 mM Pb led to the formation of calcite with a high peak intensity, and addition of 0.1 mM to 0.5 mM Pb resulted in the production of calcite and aragonite together (Supplementary Figures 3A,B). In the presence of a high concentration of Pb (1 mM), the formation of spherical vaterite was more predominant than calcite and aragonite (Figure 3F). The removal of Pb from the solution is attributed to the co-precipitation of Pb in the precipitated calcium carbonate crystal or adsorption to the crystal surface. However, in the vaterite crystal precipitated at a high concentration of Pb (1 mM), a low Pb peak was observed in the EDS graph, and the XRD and SEM-EDS analyses were not distinguished. Therefore, the concentration of Pb had little effect on the change in the geochemical properties of the medium due to the growth of microorganisms and urea decomposition, but it affected the crystallinity of precipitated calcium carbonate or the type of minerals. Similarly, the removal efficiency of Pb was higher than that of other divalent metals in previous studies of metal removal by MICP. Pb showed a removal rate of 100% in the aqueous solution using *S. pasteurii* (Mugwar and Harbottle, 2016), and 98% in the experiment using fungi (Qian et al., 2017). However, according to Mugwar and Harbottle (2016), the mechanism of Pb removal by MICP is unclear, but biological as well as abiotic mechanisms have been implicated, at least partially.

### Cadmium

Among the heavy metals used, Cd was the most toxic to microorganisms. Increasing the concentration of Cd led to inhibition of the growth of microorganisms, which corresponded to a change in the pH of the medium and the OD_600_ value (Figures 1G,H). The pH of the medium increased in the presence of 0.01 mM to 0.1 mM Cd, but not at the concentrations of 0.5 mM and 1 mM Cd, indicating the absence of active urea degradation by microorganisms (Figure 1G). The average removal rate of metal ions was 43.2% after 2 weeks of microbial reaction, under all concentrations except 1 mM Cd, and up to 60% in the case of 0.05 mM Cd (Figure 2G). However, in the case of 1 mM Cd, the amount of metal remaining was 98% or more, indicating little change (Figure 2G). The removal rate of calcium, which is directly related to the precipitate of calcium carbonate, was greater than 99% at 0.01 to 0.1 mM of Cd, whereas it was 20% and 5% at 0.5 mM and 1 mM Cd, respectively, showing a low removal rate (Figure 2H). The poor elimination rate is attributed to the growth of microorganisms and inhibition of urea decomposition, which reduced the rate of carbonate ions released and the increase in the pH of the medium, which prevented calcium carbonate formation. In the XRD pattern as well, the calcite peak with high intensity was clearly identified under a Cd concentration of 0.01 mM to 0.1 mM, but a small amount of calcite was formed with low peak intensity at 0.5 mM (Figure 3G). However, the precipitate formed at 1 mM Cd was not confirmed by XRD, but amorphous particles composed of Ca, C, and O with a size range of 0.1 to 0.2 μm were observed in SEM analysis (Figures 3G,H and Supplementary Figures 4A,B).

### Strontium

**FIGURE 4:**
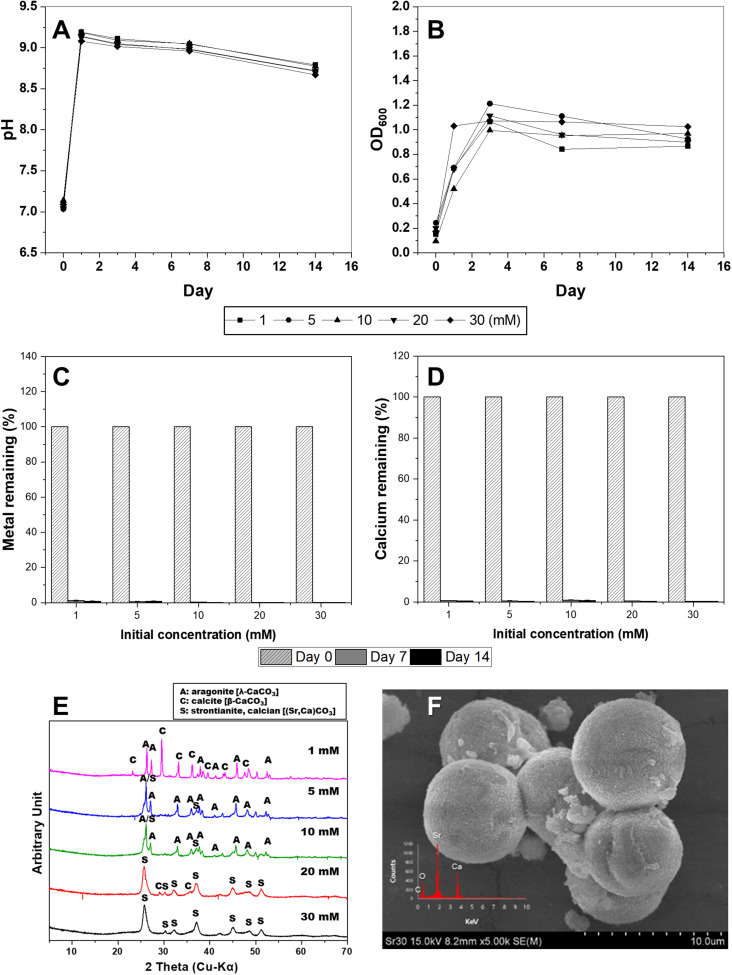
Changes in pH value **(A)**, optical density **(B)**, concentrations of Sr **(C)**, and Ca **(D)** in KCTC 3558 culture containing various strontium concentrations (mM) for 2 weeks, and X-ray diffraction spectra **(E)** of precipitates at a range of Sr concentrations and scanning electron microscopy image of strontianite **(F)**.

Unlike divalent cationic heavy metals, Sr, a radioactive element, is known to effectively sequester carbonate minerals because of its chemical similarity to calcium (Achal et al., 2012; Kang et al., 2014a; Tian et al., 2020). In this experiment, the precipitation of carbonates by *S. pasteurii* KCTC 3558was induced by varying the concentrations of Sr from 1 to 30 mM. Microbial growth was not affected by Sr concentration and significantly increased OD_600_ and pH within 3 days (Figures 4A,B). As expected, the growth of microorganisms induced the precipitation of carbonate minerals containing Sr and Ca, and most of the Sr and Ca ions in the medium were removed within a week (Figures 4C,D). The types of precipitated minerals differed according to the added Sr concentrations. Calcite and aragonite, which are calcium carbonates, were formed at a concentration of 1 mM Sr (Figure 4E). The SEM image reveals the coexistence of needle-shaped aragonite and rhombohedral calcites (Supplementary Figures 5A,B). As the concentration of Sr was increased from 5 mM to 10 mM, calcite disappeared and calcian-strontianite [(Sr,Ca)CO_3_] was formed, which was observed with aragonite (Figures 4E,F). Typically, strontianite is needle-shaped like aragonite, but with a smaller particle size and a larger aspect ratio, showing fine features. Interestingly, as the concentration of Sr increased to 30 mM, these strontianite needles were replaced by spherical particles (Figure 4 and Supplementary Figure 5). According to the recent study, bacterial somatic structure provides nucleation sites for SrCO_3_ during nucleation and growth. Also, bacterial secretions play a vital role in regulating the morphology of mineralized products (Tian et al., 2020). Therefore, in a medium containing both Sr and Ca, Sr is co-precipitated in calcium carbonate when the ratio of Sr/Ca is less than 1. As the ratio of Sr/Ca was higher than 1, Ca was co-precipitated in strontianite.

### Surface Characteristics of Calcium Carbonate Co-precipitated With Divalent Metals

**FIGURE 5:**
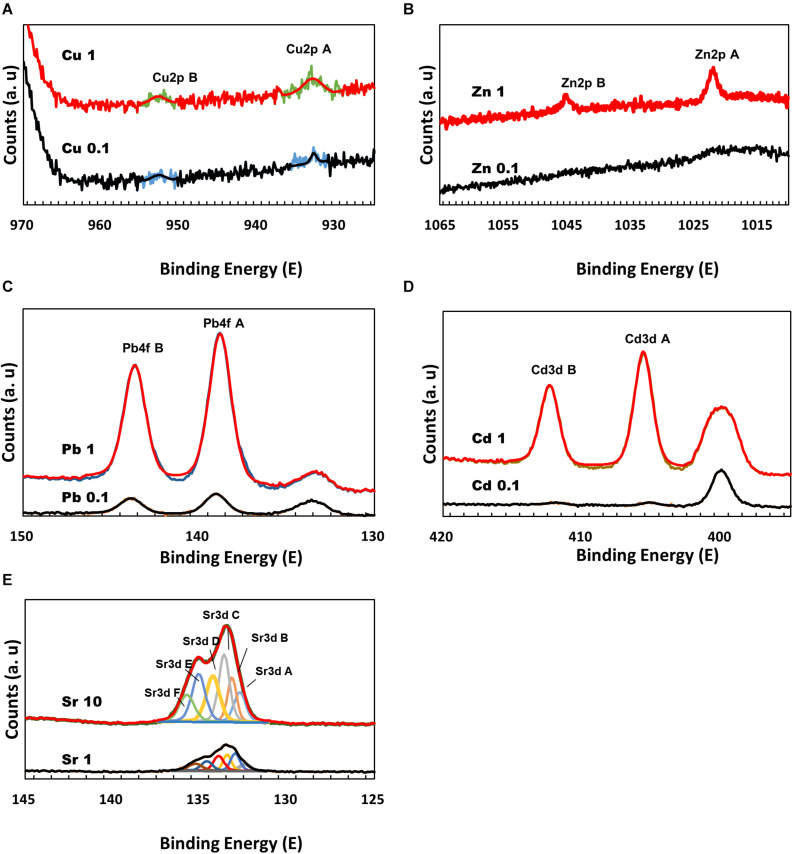
X-ray photoelectron spectroscopy spectra of calcium carbonate precipitated at a range of metal concentrations: Cu 2p **(A)**; Zn 2p **(B)**; Pb 4f **(C)**; Cd 3d **(D)**; and Sr 3d **(E)**.

To further elucidate the interaction between divalent cations and MICP, the precipitates formed during microbial growth were analyzed by XPS. The Cu 2p_3/2_ peak appeared at ∼933 eV, which is related to Cu metal, Cu (I) oxide, and Cu (II) oxide, but the differentiation of chemical state can be difficult via XPS alone. The atomic % with Cu 2p_3/2_ peak on the precipitated mineral surface was measured as 0.01 atomic % in 0.1 mM Cu and 0.06 atomic % in 1 mM Cu (Figure 5A). Zn has a Zn 2p_3/2_ peak related to ZnO observed at ∼1022 eV. In the precipitate recovered from 0.1 mM Zn, no Zn-related peak was observed; however, a related peak with 0.09 atomic % at ∼1022 eV was observed on the surface of the mineral formed with 1 mM Zn (Figure 5B). Similar to the ICP analyses in which the removal rates of Cu and Zn were low, low levels of both elements were detected (less than 0.1 atomic %) even on the surface of precipitated calcium carbonate. Pb exhibits a Pb 4f_7/2_ peak. PbO_2_ is detected at a binding energy of 137.8 eV and 2PbCO_3_.Pb (OH)_2_ at 138.4 eV. In the precipitate recovered from 0.1 mM Pb, the relevant peak was observed broadly at ∼139 eV, but on the surface of the mineral formed with 1 mM Pb, a related peak with 0.24 atomic % at ∼138.8 eV was detected with high intensity (Figure 5C). Pb contained in the calcium carbonate surface was measured close to a binding energy of ∼139 eV, suggesting that the chemical state of Pb was strongly related to PbCO_3_. The presence of Cd was confirmed by the Cd 3d_5/2_ peak at 405.1 eV. In the calcium carbonate recovered at 0.1 mM Cd, no related peak was detected, but the peak was observed on the surface of amorphous precipitates formed at 1 mM Cd (Figure 5D). Sr 3d_5/2_ peak of SrCO_3_ was detected at a binding energy of 133.4 eV (Figure 5E). XRD, ICP, SEM-EDS, and XPS analyses were confirmed that the growth of microorganisms and the formation of calcium carbonate were inhibited at 1 mM Cd, but a small amount of amorphous precipitates containing Ca and Cd were formed chemically rather than via MICP. Regardless of the types of metals, a higher metal content tended to be included on the surface of the carbonate mineral particles precipitated under high metal concentrations. Therefore, these results indicate that although the removal effect may vary depending on the type of metal, the metal ions can be co-precipitated in calcium carbonate and removed from the aqueous solution via MICP and/or chemical processes.

## Discussion

### Microbial Growth and MICP With Divalent Cations

In this study, the effects of urea-positive bacterium, *S. pasteurii* KCTC 3558, on the removal of divalent cations and the precipitation of carbonate minerals during microbial growth in aqueous solutions containing various heavy metals or a radionuclide (e.g., Cu, Zn, Pb, Cd, and Sr) were examined. As a result, the treatment with *S. pasteurii* KCTC 3558increased the pH and OD_600_ levels due to microbial growth and urea decomposition in a medium containing urea and CaCl_2_ (30 mM), creating a favorable environment for calcium carbonate precipitation. However, the rates of increase in pH and OD_600_ depended on the type and concentration of the metals in the medium. The strain was actively grown in the medium containing Cu and induced calcium carbonate precipitation. The removal of Zn was not effective during the precipitation of carbonate minerals via MICP. Unlike other metals, the increase in Pb concentration had inhibitory effect on the microbial growth and the rate of pH change. In particular, Cd was found to inhibit microbial growth at concentrations of 0.5 mM or higher. Unlike other divalent heavy metals, Sr, a radioactive element, was sequestered as carbonate minerals via MICP when it is present in high concentration due to its chemical similarity with Ca in terms of oxidation state, ionic radius, and solubility product (Goldschmidt, 1954; Achal et al., 2012., Kang et al., 2014a; Tian et al., 2020).

### Mineralogical Properties of Carbonate Minerals Formed

In addition, the types and concentrations of divalent cations influenced the shape and crystallinity of the resulting crystalline carbonate minerals (e.g., calcite, aragonite, vaterite, monohydrocalcite, and calcium-strontianite) and differences in metal removal rates. The precipitated carbonate minerals using urea-positive bacterium, *S. pasteurii* KCTC 3558, in the presence of various divalent metals and a radionuclide showed varied carbonate mineralogy, crystal morphologies, sizes, and crystallinities, which were significantly correlated with bacterial urease activity. Only calcite was formed at a Cu concentration of 0.1 to 1 mM, but the morphology varied. Calcite formed at a Zn concentration range of 0.01 mM to 0.5 mM, accompanied by aragonite formation at 0.01 mM and 0.1 mM of Zn, and vaterite formation at 0.5 mM of Zn. The inclusion of Zn (1 mM) yielded only precipitates of monohydrocalcite. Inclusion of 0.01 mM Pb led to the formation of mainly amorphous Ca-precipitates and a small amount of calcite. However, the inclusion of 0.05 mM Pb led to the formation of calcite with a high peak intensity, and addition of 0.1 mM to 0.5 mM Pb resulted in the production of calcite and aragonite together. In the presence of a high concentration of Pb (1 mM), the formation of spherical vaterite was more predominant than calcite and aragonite. The high-intensity calcite peak was clearly identified in the experiment with a Cd concentration of 0.01 mM to 0.1 mM, but a small amount of calcite was formed with low peak intensity at 0.5 mM Cd. Both calcite and aragonite were formed at a concentration of 1 mM Sr and the coexistence of needle-shaped aragonite and rhombohedral calcite was observed. As the concentration of Sr was increased from 5 mM to 10 mM, calcite disappeared and calcian-strontianite [(Sr,Ca)CO_3_] was formed, which was observed with aragonite. In previous studies, calcium carbonate formed by urease released by the *S. pasteurii* strain was reported primarily as calcite and vaterite (Sondi and Salopek-Sondi, 2005; Liang et al., 2018). Meanwhile, as shown in this experiment, aragonite was formed in a culture containing various metals (Mugwar and Harbottle, 2016). This difference is attributed to microbial urease activity and calcium uptake rate, which differ depending on the culture environment of the same microorganism, and affect calcium carbonate formation and mineralogical properties including crystal phase, size, and morphology (Liang et al., 2018).

### Removal of Divalent Cations via MICP Process

During the 2-week microbial reaction, Pb and Sr showed more than 99% removal efficiency, whereas a low removal efficiency of 30 ∼ 60% was observed at a low concentration of 0.05 mM or less of Cd, Cu, and Zn. These results show that the removal efficiency and mechanism differs depending on the type and concentration of metal ions in the MICP and affects the properties of the minerals deposited. The divalent cations may be incorporated into calcium carbonate and become a part of the crystals as calcium carbonate crystal growth continues. Among them, Pb and Sr are very similar to Ca in their chemical behavior such as ionic radius, oxidation state, and solubility (Goldschmidt, 1954). The hydrolysis of urea produces bicarbonate and ammonium, and bicarbonate participates directly in calcite precipitation, and ammonium can exchange for Sr, Ca, and Pb, resulting in enhanced susceptibility to recapture via carbonate mineral formation (Fujita et al., 2004). However, Pb^2+^ (Å = 1.19) and Sr^2+^ (Å = 1.18) ions, which have a larger ionic radius compared to Ca^2+^ (Å = 1), may be incorporated less easily in the hexagonal structure of calcite than ions smaller than Ca^2+^ (Curti, 1997; Mitchell and Ferris, 2005). So they can stabilize the crystal structure of aragonite or vaterite, which has a less compact crystal structure. According to Jiang et al. (2019), Pb had a high removal rate in the MICP process because not only bio-sorption but also abiotic and biotic precipitation occurs. This revealed that factors such as metal concentration, degree of ureolysis, and number of microbial cells would have influenced the efficiencies of abiotic precipitation, biotic precipitation, and bio-sorption, respectively (Jiang et al., 2019). Therefore, during MICP process, various divalent cations can co-precipitate with calcium carbonate through different mechanisms, which affect the type, morphology, and size of the precipitated minerals.

## Conclusion

The types and concentrations of divalent cations contributed to the carbonate mineralogy, shape, and crystallinity of various precipitated calcium carbonates and resulted in differences in metal removal rates. Pb and Sr showed more than 99% removal efficiency, whereas Cu, Zn, and Cd showed a low removal efficiency of 30∼60% at a low concentration of 0.05 mM or less. These results indicate that various divalent cations in the MICP process can co-precipitate with calcium carbonate via biologically mediated reactions which affect the mineralogy and morphology of the precipitated minerals. Microbial carbonate precipitation in the presence of divalent cations is relevant to detoxification of contaminated soils and waters as well as the synthesis of novel metal-carbonates and bio-recovery of metals and radionuclides that form sparingly soluble carbonates. Therefore, microbially induced carbonate crystallization represents an emerging and promising technology for the removal of heavy metals and radionuclides in the subsurface synthesis of carbonate minerals in the environment.

## Data Availability Statement

The original contributions presented in the study are included in the article/Supplementary Material, further inquiries can be directed to the corresponding author.

## Author Contributions

YK designed the experiments, performed analysis on all samples, interpreted the data, and wrote the manuscript. SK performed experiments and analysis and interpreted the data. YR conceived the original idea, provided critical feedback, and acted as the corresponding author. All authors contributed to the article and approved the submitted version.

## Conflict of Interest

The authors declare that the research was conducted in the absence of any commercial or financial relationships that could be construed as a potential conflict of interest.

## Supplementary Material

Supplementary Figure 1 Scanning electron microscopy image of calcium carbonate precipitated at a range of copper concentrations: Cu, 0.1 mM **(A)**; and Cu, 1 mM **(B).**

Supplementary Figure 2 Scanning electron microscopy image of calcium carbonate precipitated at a range of zinc concentrations: Zn, 0.01 mM **(A)**; and Zn, 0.5 mM **(B).**

Supplementary Figure 3 Scanning electron microscopy image of calcium carbonate precipitated at a range of lead concentrations: Pb, 0.01 mM **(A)**; and Pb, 0.1 mM **(B).**

Supplementary Figure 4 Scanning electron microscopy image of calcium carbonate precipitated at a range of cadmium concentrations: Cd, 0.01 mM **(A)**; and Cd, 1 mM **(B).**

Supplementary Figure 5 Scanning electron microscopy image of calcium carbonate precipitated at a range of strontium concentrations: Sr, 1 mM **(A)**; and Sr, 10 mM **(B).**
