# Corrigendum: Effect of Divalent Cations (Cu, Zn, Pb, Cd, and Sr) on Microbially Induced Calcium Carbonate Precipitation and Mineralogical Properties

**DOI:** 10.3389/fmicb.2021.721478

**Published:** 2021-07-12

**Authors:** Yumi Kim, Sunki Kwon, Yul Roh

**Affiliations:** Department of Earth and Environmental Sciences, Chonnam National University, Gwangju, South Korea

**Keywords:** urea hydrolysis, Sporosarcina pasteurii, bioremediation, heavy metals, bio-co-precipitation

In the original article, there was a mistake in Figure 1 as published. It was found that the graph in Figure 1H was duplicated in Figure 1C. The corrected Figure 1 appears below with the duplicated graph deleted and the missing graph of Figure 1C inserted.

**Figure 1:**
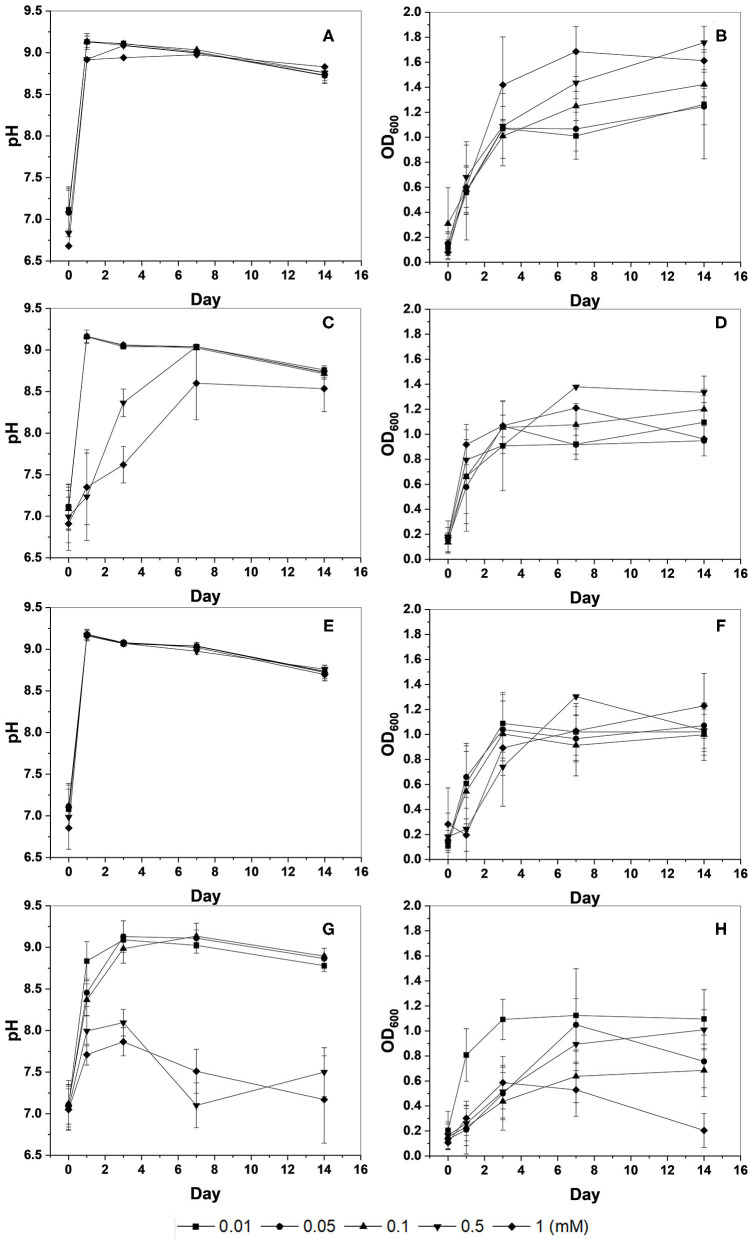
Changes in pH and optical density (OD_600_) of KCTC 3558 culture at various metal concentrations (mM): Cu **(A,B)**; Zn **(C,D)**; Pb **(E,F)**; and Cd **(G,H)**.

The authors apologize for this error and state that this does not change the scientific conclusions of the article in any way. The original article has been updated.

