# Supplementary figures and images for: Effect of Divalent Cations (Cu, Zn, Pb, Cd, and Sr) on Microbially Induced Calcium Carbonate Precipitation and Mineralogical Properties
Source: Front Microbiol. 2021 Apr 8;12:646748. doi: 10.3389/fmicb.2021.646748 (PMC8060479; doi:10.3389/fmicb.2021.646748)

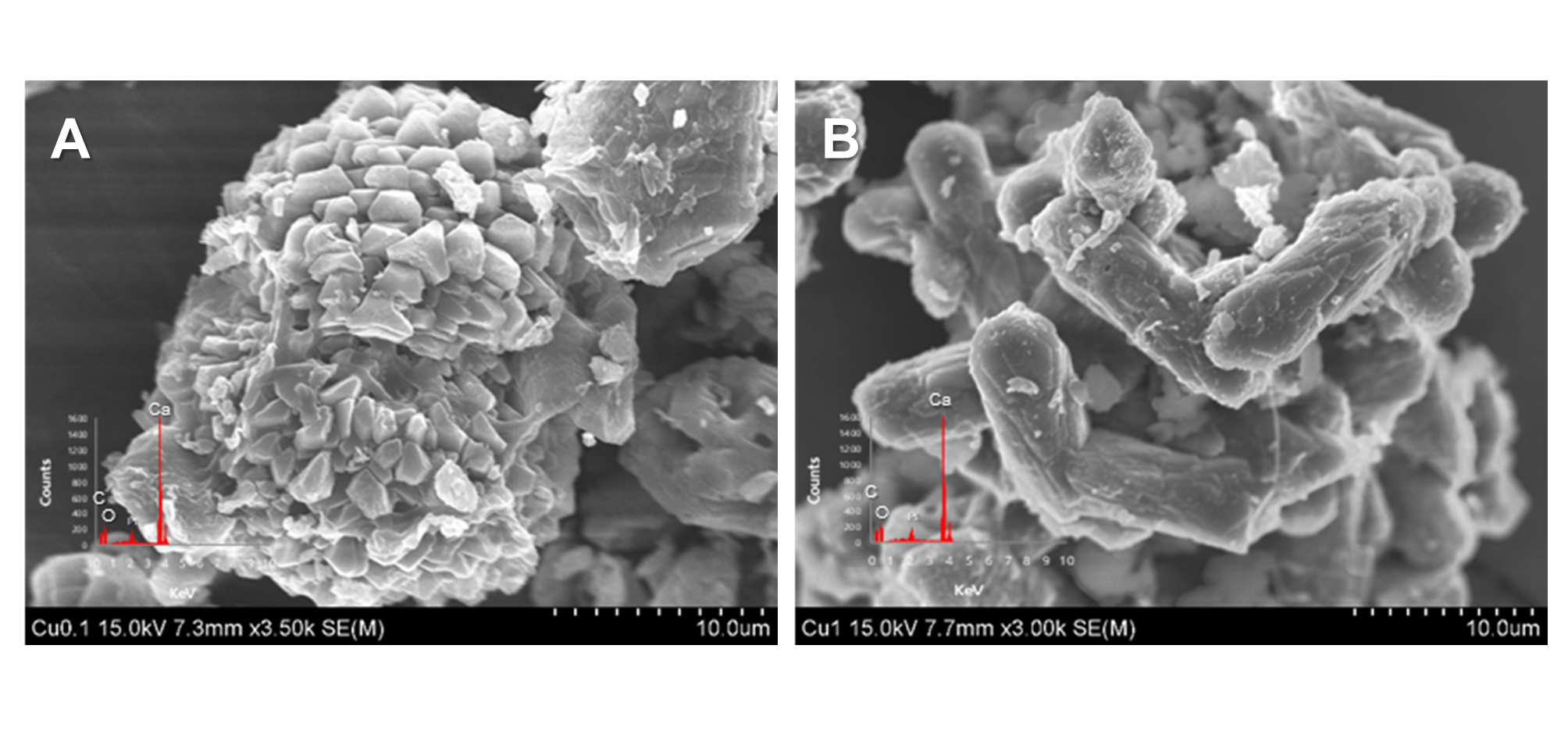

Supplement: Supplementary Figure 1 — Scanning electron microscopy image of calcium carbonate precipitated at a range of copper concentrations: Cu, 0.1 mM (A); and Cu, 1 mM (B). [file Image_1.TIF]

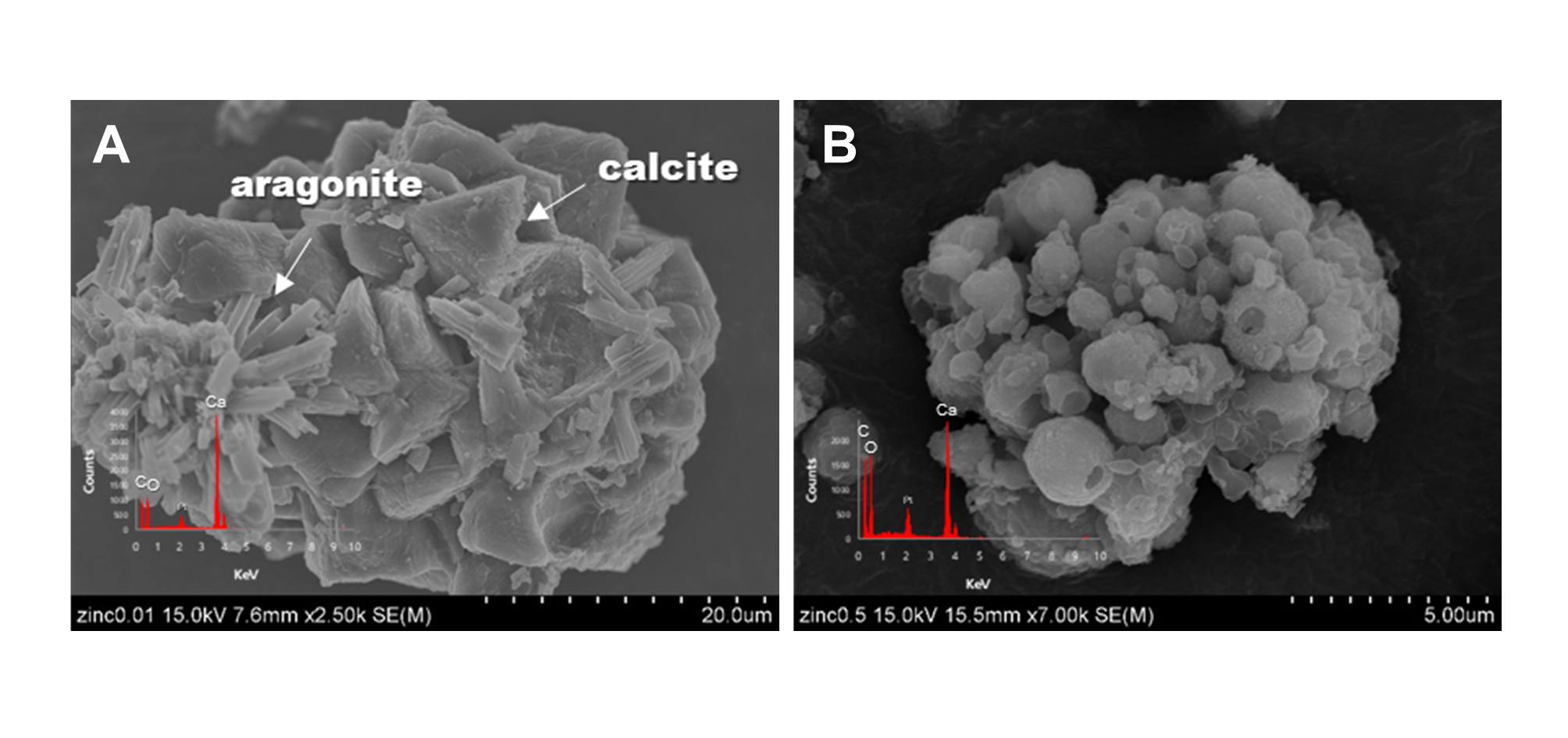

Supplement: Supplementary Figure 2 — Scanning electron microscopy image of calcium carbonate precipitated at a range of zinc concentrations: Zn, 0.01 mM (A); and Zn, 0.5 mM (B). [file Image_2.TIF]

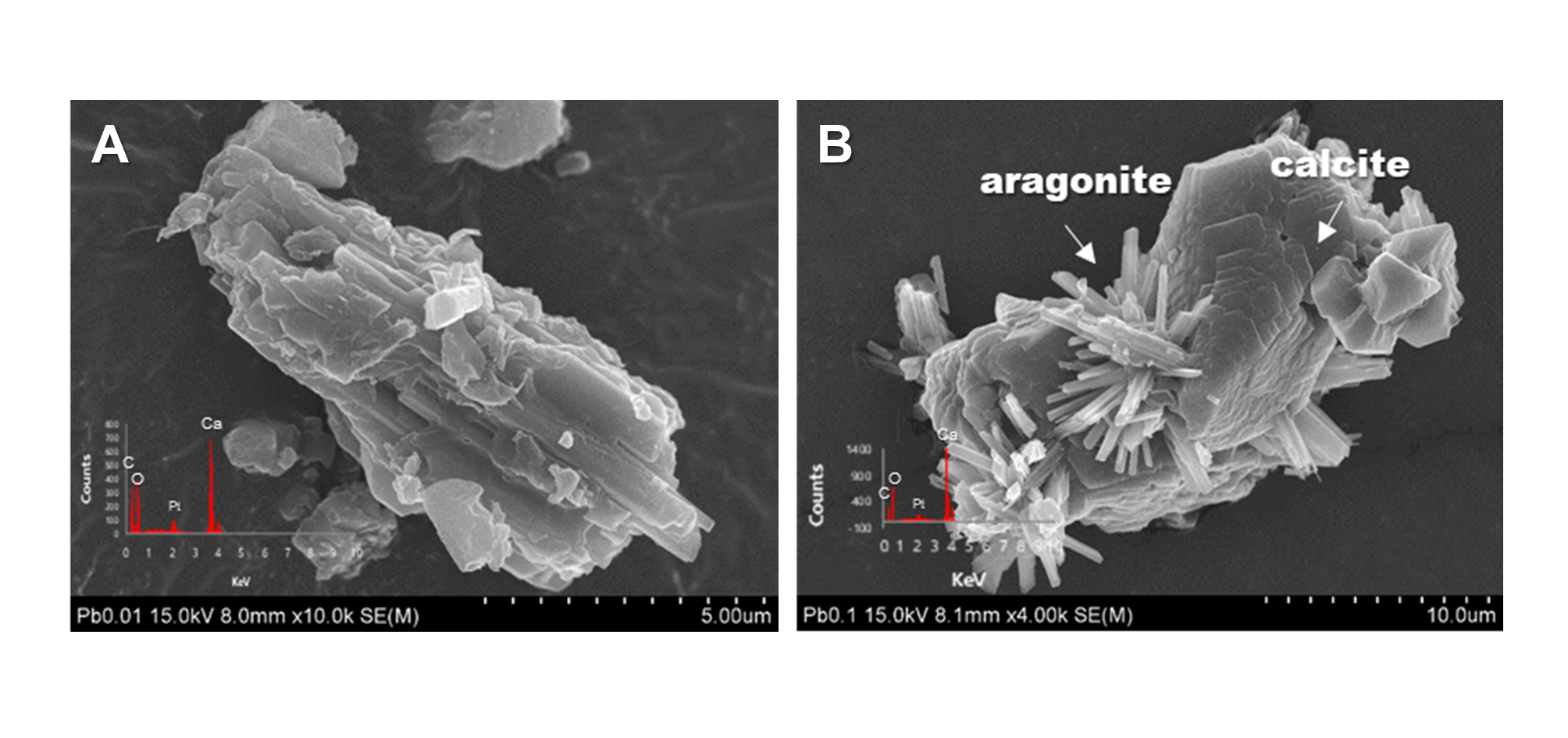

Supplement: Supplementary Figure 3 — Scanning electron microscopy image of calcium carbonate precipitated at a range of lead concentrations: Pb, 0.01 mM (A); and Pb, 0.1 mM (B). [file Image_3.TIF]

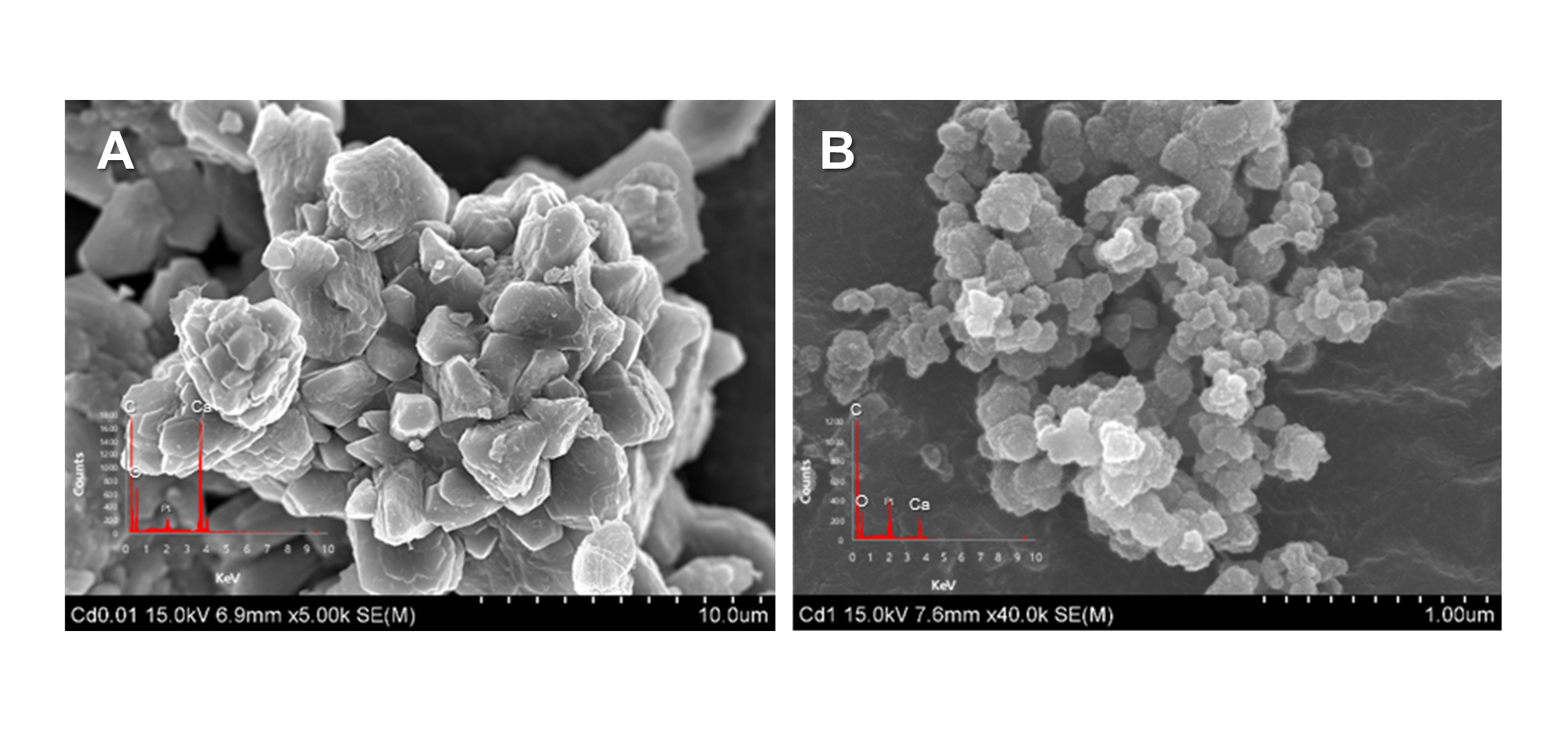

Supplement: Supplementary Figure 4 — Scanning electron microscopy image of calcium carbonate precipitated at a range of cadmium concentrations: Cd, 0.01 mM (A); and Cd, 1 mM (B). [file Image_4.TIF]

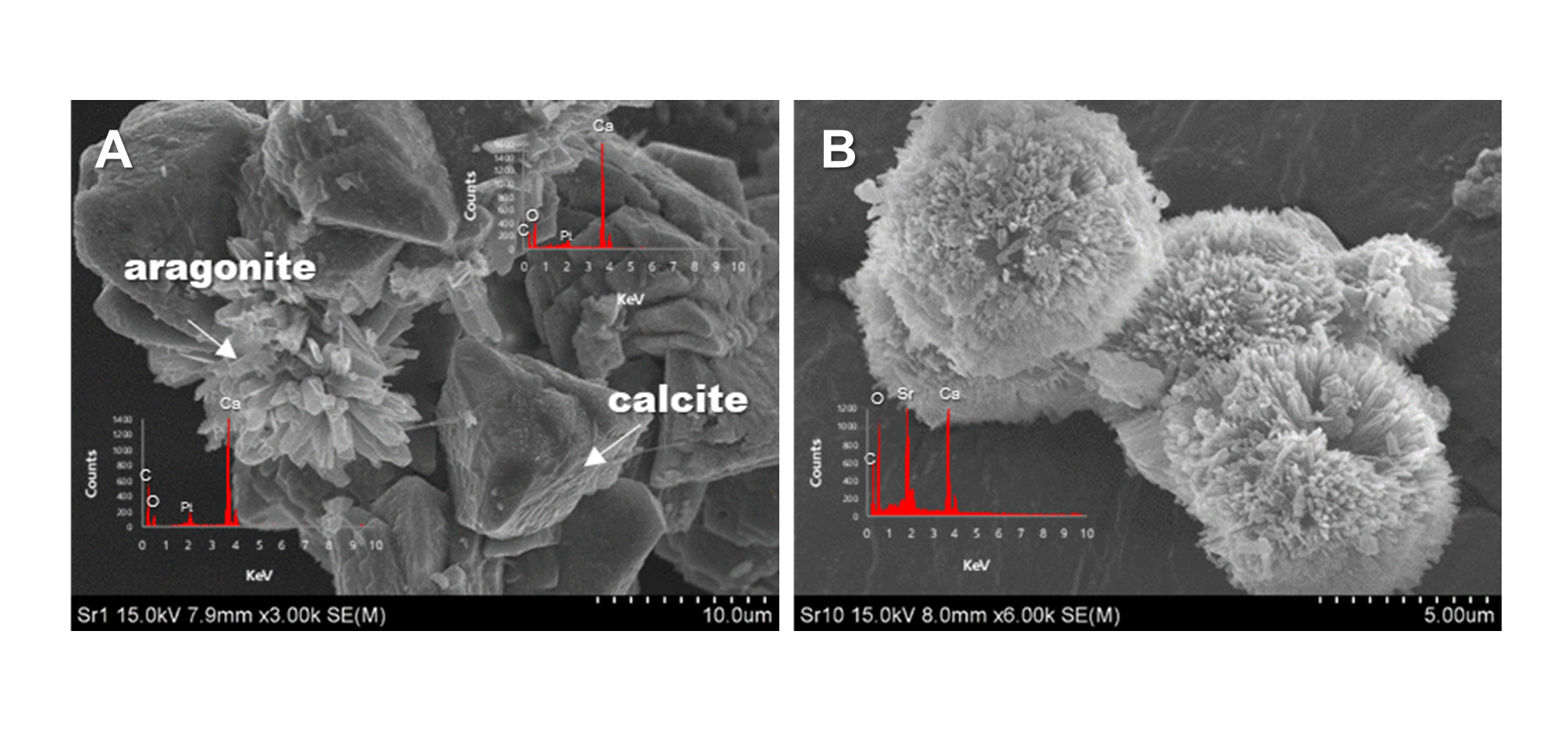

Supplement: Supplementary Figure 5 — Scanning electron microscopy image of calcium carbonate precipitated at a range of strontium concentrations: Sr, 1 mM (A); and Sr, 10 mM (B). [file Image_5.TIF]
